# Aqueous amination of track-etched polycarbonate membranes for tuneable nanochannel surface charge density

**DOI:** 10.1039/d5ra06556a

**Published:** 2025-12-05

**Authors:** Anjali Ashokan, Kamil Rahme, Subhajit Biswas, Justin D. Holmes

**Affiliations:** a School of Chemistry, University College Cork Cork T12 YN60 Ireland j.holmes@ucc.ie s.biswas@ucc.ie; b AMBER Centre, Sustainability Institute, University College Cork Cork T23 XE10 Ireland

## Abstract

Track-etched polycarbonate (PC) membranes with nanochannels are versatile materials for electrochemical, energy-harvesting, and separation applications. Precise control over their surface charge is critical, as it governs ion selectivity, electroosmotic flow, and overall ionic transport behaviour in confined nanochannels. However, environmentally friendly and scalable strategies to precisely tune their surface charge remain limited. Amination is a practical approach for PC membrane functionalisation, as it introduces protonatable amine groups that enhance the positive surface charge and enable further chemical modifications *via* mild, aqueous reactions. Here, we report a simple aqueous amination method that enables systematic control of surface charge density in PC membranes between 0.0015–0.0034 C cm^−2^. Commercial PC membranes with nominal pore sizes of 0.015, 0.05, and 0.1 µm were functionalised with a series of amines, hexamethylenediamine (HMDA), triethylenetetramine (TETA), polyethyleneimine (PEI), and glycine (Gly), through urethane-bond formation with surface carbonyl groups under mild aqueous conditions. Elemental and spectroscopic analyses confirmed efficient functionalisation and tuneable nitrogen content (9.7–22.6 at%), related to variable surface charge density, achieved by varying reaction parameters such as concentration, time, temperature, and amine type. The highest surface charge density of 0.0034 C cm^−2^ was achieved using 5% w/v TETA on PC membranes with 0.1 µm diameter. This scalable, low-energy pathway for PC membrane functionalisation is even compatible with ultrasmall pores, down to ∼15 nm. The charge densities achieved through this green aqueous functionalisation are the highest among other surface charge-tuning methods, such as plasma, ultraviolet, or polymer-grafting methods. Aqueous amination-based functionalisation is suitable for fabricating charge-tuneable, ion-selective membranes for nanofluidic energy conversion, electrochemical sensing, and other surface-charge-governed applications.

## Abstract 2

Track-etched polycarbonate (PC) membranes with nanochannels are versatile materials for electrochemical, energy-harvesting, and separation applications.

## Graphical abstract

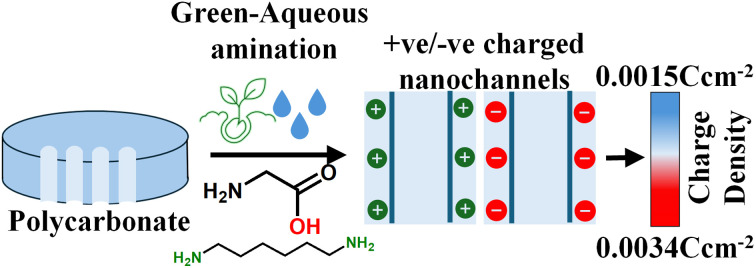

## Introduction

Track-etched polycarbonate (PC) membranes offer a unique combination of well-defined nanochannels with robust mechanical and chemical stability, making them highly suitable for various applications, including nanofiltration, gas separation, nanomaterial templating, sensing, and as separators in electrochemical and energy-harvesting devices.^1–5^ Utilisation of PC membranes in different applications requires precise control of ion transport, ion selectivity, and interfacial stability, all of which depend strongly on surface chemistry. Consequently, surface functionalisation of PC membranes has emerged as an effective strategy for tailoring surface charge density and interfacial reactivity. For instance, PC membranes modified with azo-dye groups impart a surface charge, enabling them to capture and remove pollutant azo dyes from water. Subsequent charge-assisted functionalisation further enhances the membrane's ability to reject charged contaminants such as sodium and nitrate ions.^1^ Beyond separations, precise control of PC membrane surface charge is critical in antimicrobial coatings, electrochemical sensing, and ionic energy conversion.^3,6,7^ For example, incorporating multi-walled carbon nanotubes into PC/elastomer blends enhances their electrical conductivity, transitioning from insulating to conductive behaviour observed at 10 at% ethylene propylene copolymer and 1 to 1.5 at% of nanotubes.^8^ Thus, surface engineering through chemical modification is a promising strategy for optimising PC membrane performance.

A wide range of functionalisation strategies has been developed for PC membranes,^7,9–11^ yet their intrinsic hydrophobicity, thermal stability, and low surface energy pose significant challenges.^12,13^ Most established approaches, such as plasma modification,^14^ polymer grafting,^14,15^ atomic layer deposition (ALD),^16^ and UV light exposure,^17^ either require specialised equipment, high energy input, or generate unstable surfaces. For example, UV irradiation risks chain scission of the PC backbone,^18^ plasma processes often lead to shallow or non-uniform functionalisation, and ALD methods, while precise, are costly and limited in scalability. More aggressive acid or high-temperature treatments can introduce hazardous by-products and compromise mechanical integrity. These constraints limit the broader adoption of such strategies in the scalable fabrication of nanofluidic devices from functionalised PC membranes.

Recent studies have therefore focused on more accessible functionalisation strategies that operate under aqueous or low-energy conditions. An Delinder *et al.*^19^ reported a benign aqueous amination of polycarbonate films using diamines under mild conditions, achieving covalent surface amination without polymer degradation. Layer-by-layer (LBL) deposition of polyelectrolytes, composed of amino acid sequences, provides another route to functionalise PC membranes.^20^ Radical-initiated polymerisation of acrylic acid (AAc), acrylamide (AAm), and methyl methacrylate (MMA) has also been used to fabricate hydrogel-hybrid PC membranes with improved ion transport, selectivity, and osmotic energy conversion properties.^3^ More recently, Rahimnejad *et al.*^21^ functionalised porous PC membranes by incorporating TiO_2_ nanoparticles *via* glutaraldehyde crosslinking, enhancing hydrophilicity and water flux under non-extreme conditions. Beyond PC systems, several other studies highlight a growing shift toward sustainable polymer-grafting and low-energy functionalisation approaches on membrane surfaces to enhance performance.^22–24^

In addition, emerging membrane technologies have demonstrated diverse strategies for sustainable or performance-driven surface modification. For instance, Na-bentonite-embedded MXene composite membranes prepared *via* hydrothermal-vacuum assembly exhibit durable antifouling properties and enhanced chemical stability for oil–water separation.^25^ Likewise, methanetetrayltetrakis(benzene-1,2-diamine)-based thin-film composite membranes fabricated through interfacial polymerisation achieve defect-free selective layers and excellent antibiotic desalination performance.^26^ Furthermore, molecularly imprinted polymeric membranes incorporating dansyl-derived fluorescent monomers have been explored for selective detection of lipopolysaccharides, demonstrating biocompatible, reusable sensing functionality.^27^ Collectively, these developments highlight the growing emphasis on environmentally conscious, versatile membrane design strategies that align with the fully aqueous functionalisation approach presented here.

Despite these advances, most approaches still rely on multistep syntheses, organic solvents, or composite assemblies and achieve only limited charge densities. In contrast, charge incorporation within small pores (∼15 nm) remains largely unexplored. A mild, aqueous, and scalable approach capable of producing both positively and negatively charged PC membranes while preserving pore morphology is therefore highly desirable. This work demonstrates a fully aqueous, substrate-compatible functionalisation strategy that enables precise modulation of surface charge density in PC membranes. To achieve this, an aqueous amination strategy for commercially available track-etched PC membranes (8–25 µm thick, pore diameters 15–100 nm) was developed. By reacting surface carbonyl groups with amine-containing molecules, hexamethylenediamine (HMDA), triethylenetetramine (TETA), polyethyleneimine (PEI), and glycine (Gly), through urethane-bond formation, tuneable nitrogen incorporation (9.7–22.6 at%) and corresponding surface-charge densities (0.0015–0.0034 C cm^−2^) were achieved. This aqueous process proceeds under mild conditions, avoids the use of organic solvents, and remains compatible with ultrasmall pores (∼15 nm). The resulting charge-tuneable PC membranes provide a scalable, low-energy platform for ion-selective transport, nanofluidic energy conversion, and other surface-charge-driven electrochemical applications.

## Materials and methods

### Materials

The following materials were used in this study: Whatman® Isopore™ PC membranes (25 mm diameter, 0.1 µm nominal pore size, PC100), Cytiva Whatman® Isopore™ Nucleopore track-etched membranes (13 mm diameter, 0.015 µm nominal pore size, PC15), and Cytiva Whatman® Nucleopore track-etched membranes (90 mm diameter, 0.05 µm nominal pore size, PC50), all purchased from Merck. Hexamethylenediamine (HMDA), triethylenetetramine (TETA), glycine (Gly), polyethyleneimine (PEI) of molecular weight 0.8 KD and sodium hydroxide (ACS reagent, ≥97.0%, pellets) were obtained from Sigma-Aldrich. Milli-Q water was used as the sole solvent in all experiments.

### Aqueous functionalisation of polycarbonate membranes

PC membranes of varying pore sizes (PC15, PC50, and PC100) and thicknesses were aminated using a one-pot approach. For each functionalisation process, PC membrane samples were cut to approximately 1 cm^2^. In a typical procedure, the membranes were placed in a glass container and dried in a vacuum oven for 1 h to remove residual moisture and air from the pores. The membranes were then immersed in aqueous amine solutions of defined concentrations (Table 1). Four amine-containing molecules (HMDA, TETA, PEI, and Glycine) with distinct chain lengths and nitrogen functionalities were selected to tune the surface charge density of PC membranes. To enhance pore infiltration and eliminate trapped air bubbles, the immersed membranes were subjected to vacuum degassing for an additional 40–60 min. After vacuum treatment, the solutions were sealed to prevent atmospheric exposure and placed on a shaker (90–120 rpm). Samples were then reacted under the following conditions (Table 1).

**Table 1:** Reaction conditions for aqueous amination of PC membranes with different functionalising agents

Functionalising agent	Concentration (% w/v)	Temperature	Duration
HMDA	1–2.5	RT	72 h
TETA	1–5	RT + 70 °C	RT(74 h) + 70 °C(2 h)
TETA (high conc.)	5	70 °C	2 h
PEI (0.8 KD)	5–10	RT + 70 °C	RT(72 h) + 70 °C(2 h)
PEI (0.8 KD) (high conc.)	10	70 °C	2 h
Glycine	5	70 °C	22 h

After reaction, the functionalised PC membranes were rinsed thoroughly with Milli-Q water to remove unreacted species, then dried at 60 °C and stored in sealed containers until characterisation. The chosen temperature range and other reaction conditions were explored to ensure maximum grafting while maintaining mechanical integrity.

### Rationale for the selected amine molecules

**Fig. 1:**
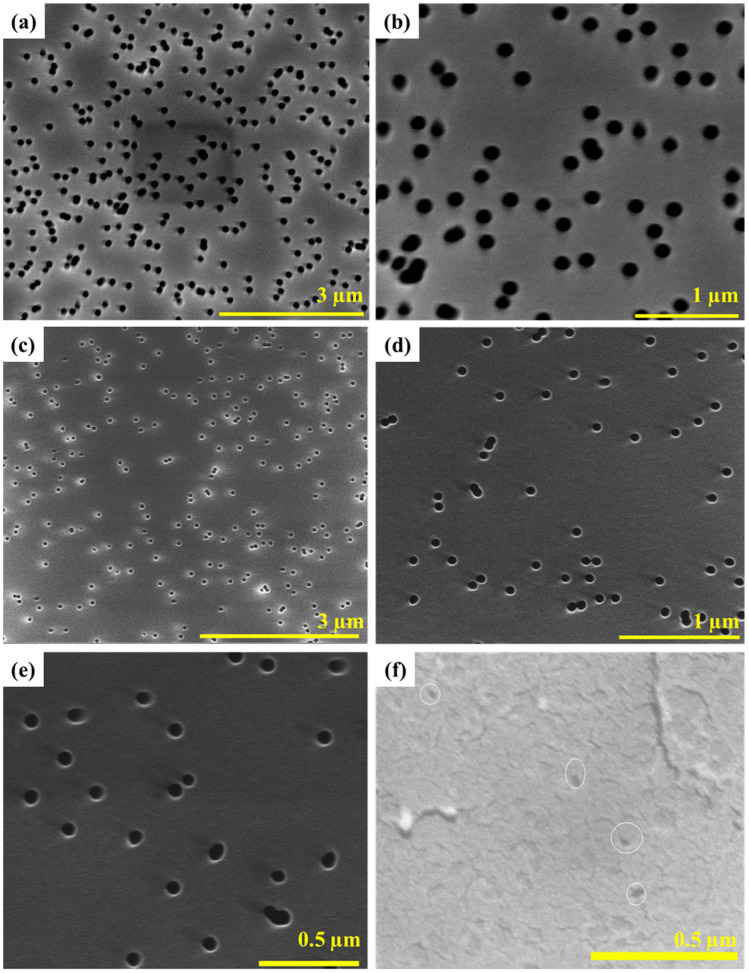
SEM images of track-etched polycarbonate membranes: (a) and (b) Isopore™ Merck Millipore, pore size 0.1 µm; (c)–(e) Isopore™ Merck Millipore, pore size 0.05 µm; (f) Isopore™ Merck Millipore, pore size 0.015 µm.

Hexamethylenediamine (HMDA) is a small aliphatic diamine containing two primary amine groups separated by a flexible C_6_ chain (Fig. 1(a)), allowing efficient nucleophilic attack on the carbonate carbonyl while maintaining molecular mobility within nanochannels.^19^ Surface functionalisation proceeds *via* nucleophilic substitution of the carbonate carbonyl on the PC backbone by the primary or secondary amine, producing a urethane (carbamate) linkage; see the reaction schemes given in the results and discussion section and general reaction equation shown below (eqn (1)).

1 PC–O–CO–O– + H_2_N–R–NH_2_ → PC–O–CO–NH–R–NH–CO–O–PC + ROH

This reaction introduces surface amino groups that can protonate to yield positively charged –NH_3_^+^ species under aqueous conditions. Preliminary tests revealed that high amine concentrations or prolonged exposure cause carbonate chain cleavage, leading to membrane disintegration for HMDA reaction. Hence, a small molecule similar to HMDA, but with higher amine density, triethylenetetramine (TETA), was chosen. TETA consists of four amine groups (secondary and primary amines with six carbon atoms) that increase the nitrogen content per molecule, providing a higher density of potential protonation sites and, therefore, a stronger positive surface charge. Polyethyleneimine (PEI, *M*_w_ ≈ 0.8 kDa) is a branched polymer bearing a high ratio of primary, secondary, and tertiary amines. It was explored to evaluate the upper limit of achievable surface charge density and to assess diffusion constraints of macromolecules within confined pores. Glycine (Gly), the simplest amino acid containing both amine and carboxylic acid functionalities, was chosen to generate negatively charged surfaces *via* its deprotonated –COOH group.

### Characterisation

Scanning electron microscopy (SEM) analysis was performed using a Quanta SEM 650 operating at 20 kV, equipped with an energy-dispersive X-ray (EDX). Samples were gold-coated to prevent charging effects. Surface wettability was evaluated using an electronic goniometer (Ossila Ltd) based on the sessile drop method, where ∼5 µL of deionised water was deposited onto each sample using a fixed syringe needle, and contact angle (*θ*) measurements were averaged over three independent measurements per sample. Attenuated total reflectance Fourier transform infrared spectroscopy (ATR-FTIR) was conducted using a PerkinElmer Spectrum Two FT-IR spectrometer with a resolution of 1 cm^−1^ over a scanning range of 400–4000 cm^−1^. X-ray photoelectron spectroscopy (XPS) measurements were performed on a Kratos Axis Ultra spectrometer equipped with a monochromatic Al K_α_ (1486.58 eV) X-ray source. Full survey spectra were collected using an analyser pass energy of 160 eV and a step size of 1 eV. In comparison, high-resolution spectra were obtained at a pass energy of 20 eV with a step size of 0.05 eV for detailed elemental analysis. Spectra were recorded in the normal-to-surface direction, with the C 1s peak at 284.8 eV serving as a charge reference, with an analysis area of approximately 1 mm^2^ and a depth of ∼10 nm. Data processing involved Shirley-type background correction and peak fitting using a synthetic-peak model with mixed Gaussian–Lorentzian functions. At the same time, elemental quantification was based on relative sensitivity factors from the Casa XPS library incorporating Scofield cross-sections, with high-resolution spectra analysed to determine elemental composition.

### Estimation of grafting density (mol cm^−2^) and charge density (C cm^−2^)

Grafting density and charge density after functionalisation were estimated for the atomic% (at%) of N. The N at% was determined directly from XPS. This value represents the percentage of nitrogen atoms relative to all atoms detected within the surface-sensitive sampling depth (∼10 nm). Nitrogen atoms per molecule were estimated from the chemical structure of the grafted molecule or polymer. For example, HMDA has two amine groups, resulting in two nitrogen atoms per molecule. In contrast, TETA and PEI have 4 and 14 nitrogen atoms per molecule, respectively. To convert the N at% to N atoms per unit area (N atoms cm^−2^), *i.e.* an absolute surface number density, we used eqn (2):2
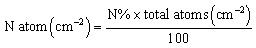
In this equation, N% is measured from XPS. Total number of atoms is calculated from bulk atom density of polycarbonate (∼9.4 × 10^22^ atoms cm^−3^) and XPS sampling depth (∼10 nm = 1 × 10^−6^ cm). Thus, the total atoms per unit area: total atoms cm^−2^ = (9.4 × 10^22^) × (1 × 10^−6^) = 9.4 × 10^16^ atoms cm^−2^.

Grafting density (*ρ*, mol cm^−2^) after functionalisation of PC membranes was calculated from eqn (3).3
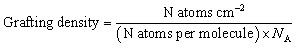
where, *N*_A_ is Avogadro number (6.022 × 10^23^ mol^−1^). Assuming each grafted nitrogen atom contributes to surface charge, the charge density (*σ*, C cm^−2^) was estimated with eqn (4).

4 Charge density = N atoms × *e*

where, *e* = 1.6 × 10^−19^ C.

## Results and discussion

Commercially available PC membranes with thicknesses of 8 to 25 µm and nominal pore diameters of 15 nm (PC15), 50 nm (PC50), and 100 nm (PC100) were used in this study (Fig. S1 in the SI presents digital images of the membranes). Surface SEM characterisation and analysis (Fig. 1 and S2 in the SI, respectively) confirmed the pore sizes, with measured diameters closely matching the supplier's specifications. The membranes exhibited pore coverage of 10–14.4%.

To modify the PC membranes, which have varying nominal pore sizes, we used two amino-group-containing small organic molecules: hexamethylenediamine (HMDA) and triethylenetetramine (TETA). Additionally, we employed a branched polymer, polyethyleneimine (PEI), to impart a positive charge to the surfaces of the PC membranes. In contrast, to compare the functionality of different organic groups, PC membranes were also functionalised with glycine to graft negative surface charges *via* the carboxylic group. The amine reacted with the carbonyl group of the PC surface by nucleophilic substitution to functionalise the membrane with amine groups.^19^ Moreover, to modify the grafting density of these amines onto PC membranes, the concentration of molecules was varied in water. This systematic selection of amine molecules establishes a direct link between different amine molecular structures (chain length, amine functionality, molecular size) and the resulting surface charge density, enabling mechanistic insights into charge–structure relationships in aqueous PC membrane functionalisation.

### Functionalisation of PC membrane with small molecules for positive charge

**Fig. 2:**
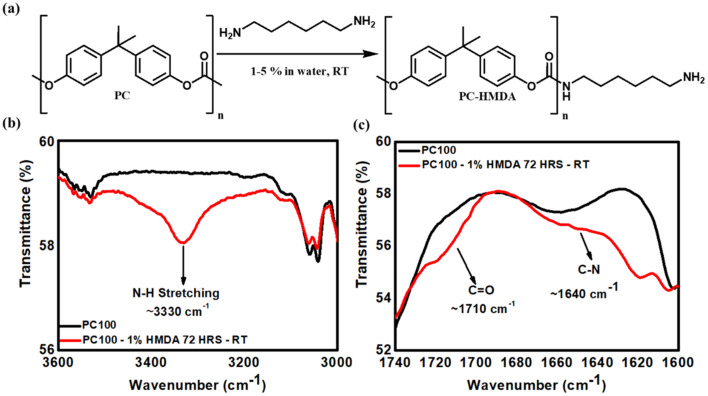
(a) Schematic representation of the chemical derivatisation of PC membrane with hexamethylenediamine (HMDA). FTIR spectra of PC100 membranes before and after functionalisation with (HMDA) 1% w/v, highlighting (b) the region 3000–3600 cm^−1^ where the N–H stretching signal from HMDA appears, and (c) the carbonyl group (C=O) region 1600–1740 cm^−1^, showing slight shifts indicative of urethane bond formation between HMDA and PC.

Hexamethylenediamine (HMDA) was selected as the model diamine to investigate the feasibility of covalent amination of polycarbonate (PC) membranes under purely aqueous conditions. HMDA contains two terminal primary amine groups separated by a flexible six-carbon chain, providing both high nucleophilicity and chain mobility to facilitate attack on the electrophilic carbonate carbon of the PC backbone.^19^ The reaction proceeds *via* nucleophilic substitution of the carbonate carbonyl, leading to urethane (carbamate) bond formation and the introduction of surface amino functionalities (Fig. 2(a)). This chemistry was chosen because it represents the most straightforward aqueous route to generate a positively charged PC surface while maintaining molecular diffusion compatibility with sub-100 nm pores.

PC membranes of nominal pore diameters 15–100 nm were reacted with aqueous HMDA solutions (1–5% w/v) at room temperature (RT) for 72 h. The grafting density was controlled by adjusting amine concentration. At HMDA loadings above 2.5% w/v, the membranes gradually lost mechanical integrity, indicating that excessive nucleophilic attack led to scission of the carbonate chain. Consequently, 1–2.5% w/v was identified as the optimal range balancing reactivity and polymer stability. Similar structural degradation beyond 2.5% w/v was observed in both PC15 and PC50 membranes, suggesting that reaction kinetics are primarily governed by chemical reactivity rather than pore geometry.

Pristine PC membranes exhibited characteristic peaks of polycarbonate (see Fig. S3, in SI): C–H aromatic ring deformations around 3000 cm^−1^; C=O groups at 1775 cm^−1^; C=C vibrations at 1507 cm^−1^; asymmetric O–C–O in the range of 1240–1142 cm^−1^ and symmetric O–C–O near 1015 cm^−1^.^28^ FTIR spectra of PC100 given in Fig. 2(b) and (c), and PC50 and PC 15 presented in Fig. S4, in SI, provide direct evidence of covalent HMDA incorporation for PC membranes with different nanopore diameters. The results clearly show a new band from the N–H stretch of a primary amine at 3330 cm^−1^ (Fig. 2(b), S4 (a) and (c) SI), which is absent in the pristine PC spectrum. The new peaks at 3330 (N–H stretching of primary amine), 1710 (urethane C=O stretching), and 1640 cm^−1^(C–N stretching) that were assigned to the groups of –OC(=O)NH–, confirmed the attachment of diamine. Notably, such clear urethane and amine features were not observed by van Delinder *et al.*,^19^ who used a similar aqueous amination at 1% w/v for 72 h, highlighting that the optimised conditions used here enhance reaction efficiency even under mild conditions. Furthermore, contact-angle measurements complement the FTIR results, with an increase from 71.7° for untreated PC100-Blank to 91.7° for PC100-HMDA. Similarly, for PC50, the contact angle rose from 67.8° to 95.3°, and for PC15, it increased from 64.5° to 81.2° (see Table S1 in the SI) upon HMDA functionalisation. The increase in the hydrophobic character of the membrane is attributed to the alkyl unit of hexamethyldiamine, comprising six –CH_2_- groups, which may confer some hydrophobicity to the membrane. Such wettability modulation demonstrates that surface polarity can be tuned by the molecular structure of the grafted amine, a key design parameter for controlling interfacial charge density and ion-transport behaviour.

**Fig. 3:**
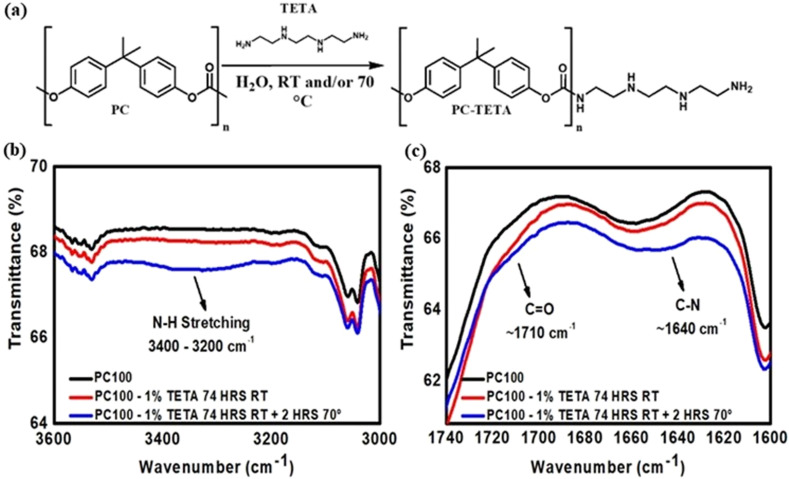
(a) Schematic representation of the chemical derivatisation of PC membrane with triethylenetetramine (TETA). FTIR spectra of PC100 membranes before and after functionalisation with (TETA) 1% w/v, highlighting (b) the region 3000–3600 cm^−1^ where the N–H stretching signal from TETA appears, and (c) the carbonyl group (C=O) region 1600–1740 cm^−1^, showing slight shifts indicative of urethane bond formation between TETA and PC.

As PC degraded under high loading of HMDA, another small amine-containing molecule, with a higher amine density than HMDA, triethylenetetramine (TETA) was used for functionalisation (Fig. 3(a)). TETA contains four nitrogen sites (two primary and two secondary amines) distributed along a flexible ethylene chain, which provides a higher density of reactive sites and greater potential for protonation upon grafting. The initial reaction of PC with TETA appeared ineffective at RT, as indicated by FTIR (Fig. 3(b) and (c)) of the PC membranes after functionalisation. Contact angle measurements (see Table S2 in SI) did not show any significant change before or after functionalisation with TETA at RT. This is likely due to the steric crowding of neighbouring amines and the lower nucleophilicity of secondary amines. Hence, to overcome this, an additional reaction step was introduced, elevating the reaction temperature to 70 °C for 2 h after the reaction at 74 h at RT. This short heating period substantially enhanced amination efficiency, as confirmed by FTIR and surface wettability measurements.

Fig. 3(b) and (c) and present the FTIR results for untreated PC100-Blank, unreacted PC100-TETA 1% w/v at RT, and PC100-TETA 1% w/v solution, after heating for 2 h. PC-Blank and PC-TETA at RT showed similar spectra with no significant changes. At the same time, a new band from the N–H stretch of a primary and secondary amine in TETA between 3200–3400 cm^−1^ and urethane-related peaks at 1600–1720 cm^−1^ (Fig. 3(b) and (c)) were observed for PC-TETA at 70 °C. The pronounced increase in the intensity of the N–H stretching band upon heating indicates successful and efficient grafting of TETA *via* thermal treatment. Surface wettability changes further corroborate the enhanced grafting of TETA. The contact angle decreased from 64.6° (PC100-Blank) to around 56.4° (PC-TETA), indicating increased surface polarity arising from the introduction of four hydrophilic amino groups per TETA molecule. Comparable trends were observed across other pore sizes, confirming the uniformity of TETA functionalisation. For PC50, the contact angle decreased from 67.8° to 55.9°, and for PC15, from 64.5° to 51.4°, suggesting that the functionalisation procedure is effective and compatible even with ultrasmall pores. To further modulate grafting density, the TETA concentration was increased from 1% to 5% w/v, then to 10% w/v. Building on earlier findings that thermal treatments improved the functionalisation efficiency for 1% w/v TETA, the 5% w/v TETA reaction was carried out directly at 70 °C for 2 h, thereby eliminating the initial functionalisation step at RT.

### Quantitative analysis of functionalised PC membranes with positive charge

**Fig. 4:**
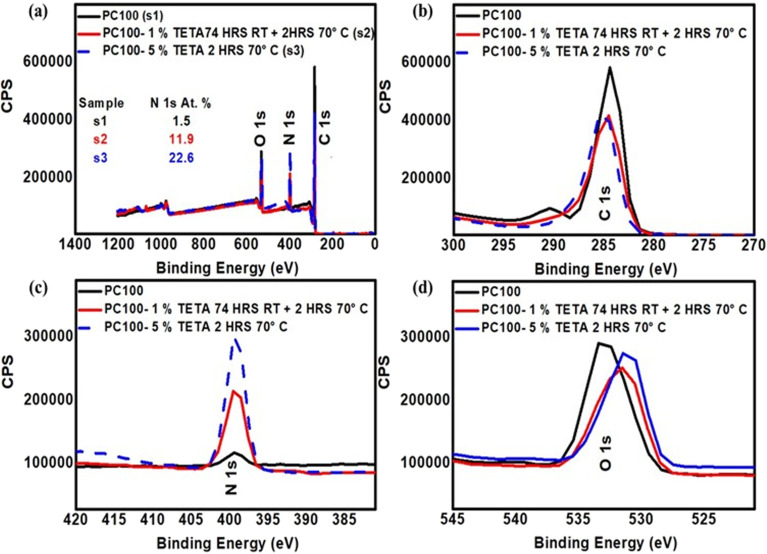
(a) XPS survey spectra of PC100 membranes before and after functionalisation with 1% w/v TETA (74 h at RT + 2 h at 70 °C) and 5% w/v TETA (2 h at 70 °C), with N 1s at% indicated. High-resolution spectra of (b) C 1s (270–300 eV), (c) N 1s (380–420 eV), and (d) O 1s (520–545 eV) regions show increased N 1s signal and shifts in C 1s and O 1s peaks, confirming successful TETA grafting onto PC membranes.

To quantitatively confirm the covalent attachment of amine molecules and to evaluate the effects of small-molecule concentration and molecular structure, XPS was performed on HMDA- and TETA-functionalised membranes. Fig. 4 and S5 (see SI), show the XPS survey and high resolution (C, O and N) spectra for representative PC100-TETA and PC100-HMDA, respectively, while Tables S3 and S4 (see SI) give the summary of elemental percentage quantification of PC100, PC50 and PC15 before and after functionalisation. Since each grafted amino group contributes one nitrogen atom, the nitrogen atomic percentage (N at%) calculated from XPS provides a direct measure of grafting density and the potential surface-charge density.

**Fig. 5:**
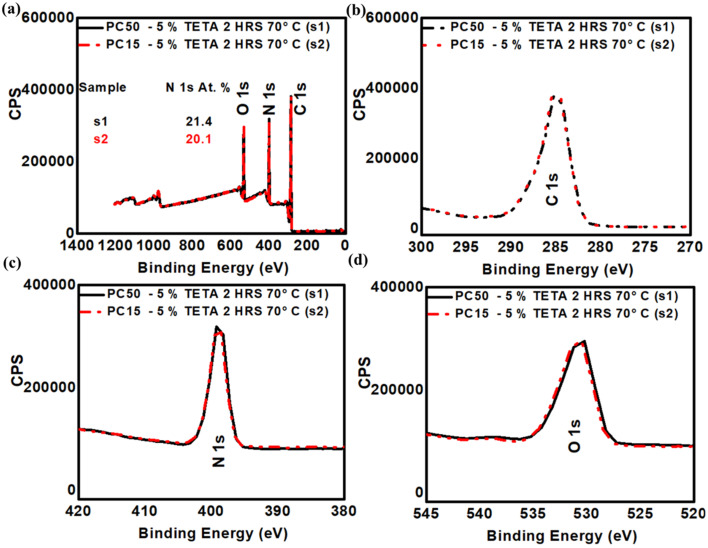
(a) XPS survey spectra of PC50 and PC15 membranes before and after functionalisation with 5% w/v TETA (2 h, 70 °C), with the corresponding N 1s atomic percentages indicated. High-resolution XPS spectra of the (b) C 1s (270–300 eV), (c) N 1s (380–420 eV), and (d) O 1s (520–545 eV) regions.

For PC100 HMDA functionalisation at 1% w/v produced 9.7 at% N (see Fig. S5, in SI), while increasing the concentration to 2.5% w/v raised the nitrogen content to 12.5 at%. This monotonic rise indicates that higher HMDA loading promotes a greater density of amine terminations, up to the point at which excess nucleophilic attack degrades the carbonate backbone (as evidenced by visual degradation). The results confirm that small, linear diamines can achieve significant surface modification under mild conditions. TETA exhibited even greater nitrogen enrichment at the surface, consistent with its higher density of reactive amine sites. A 1% w/v TETA treatment yielded 11.9 at% N, already exceeding the at% values for nitrogen obtained with HMDA (Fig. 4). Increasing the concentration to 5% w/v and introducing a short heating step (70 °C, 2 h) elevated the nitrogen content further to 22.6 at%. Comparable nitrogen levels were achieved for PC50 and PC15 membranes at 5% w/v, as illustrated in Fig. 5. The XPS survey and high-resolution (C 1s, O 1s, N 1s) spectra confirm successful amine grafting, with the PC50 membrane exhibiting 21.4 at% N and PC15 showing a slightly lower yet substantial 20.1 at% N. These results collectively validate the strong affinity of TETA grafting and highlight its efficiency in producing highly aminated membranes across sub-100 nm pore sizes. It is important to note that the HMDA functionalisation was performed entirely at RT, whereas the TETA treatment included an additional thermal step (2 h at 70 °C).

To assess whether similar thermal conditions would enhance HMDA grafting, the same thermal protocol was applied to HMDA functionalisation. However, even for 1% w/v HMDA solutions, these membranes degraded, reinforcing that the multi-amine structure of TETA enables higher reactivity without extensive chain scission. To further increase N beyond the 22.6% obtained, the reaction duration with TETA was extended from 2 to 24 h at 70 °C. However, when the PC membranes were heated to 70 °C for 24 h with TETA, they degraded. Similarly, increasing the TETA solution concentration to 10% w/v also resulted in membrane degradation, even after a 2 h reaction at 70 °C. This observation is consistent with over-substitution of carbonate linkage at high TETA concentration. Overall, the XPS data clearly reveal that increasing the number of amine groups per molecule enhances surface nitrogen incorporation, and mild thermal activation improves grafting efficiency, especially for TETA. However, there exists a threshold of 5 w/v % TETA beyond which excessive nucleophilicity induces degradation.

### Functionalisation of the PC membrane with a polymer for positive charge

Having established the reactivity trends and parameters for small amines, a large polymeric amine, polyethyleneimine (PEI, *M*_W_ ≈ 0.8 KD), was examined for higher nitrogen grafting. PEI is a well-established polyelectrolyte composed of primary, secondary, and tertiary amines, providing a high amine-to-carbon ratio and strong affinity for carbonyl-containing polymers. Its branched structure offers multiple reactive sites but also introduces steric hindrance that can restrict diffusion into nanoscale pores. Therefore, PEI serves as a useful probe to evaluate how polymer chain size and conformational freedom influence amination efficiency within the confined geometry of track-etched PC membranes.

**Fig. 6:**
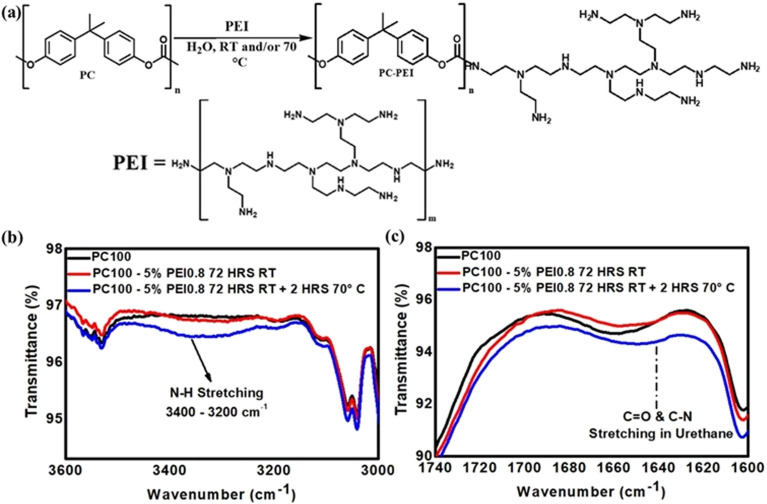
(a) Scheme representation of the chemical derivatisation of PC membrane with polyethyleneimine (PEI 0.8 KD-branched). FTIR spectra of PC100 membranes before and after functionalisation with (PEI) 5% w/v, highlighting (b) the region 3000–3600 cm^−1^ where the N–H stretching signal from PEI appears, and (c) the carbonyl group (C=O) region 1500–1700 cm^−1^, showing slight shifts indicative of urethane bond formation between PEI and PC.

The covalent bonding of PEI to the PC surface proceeds through nucleophilic attack of the polymer's amine groups on the carbonate carbonyl, leading to urethane linkage formation, as illustrated schematically in Fig. 6(a). The reaction conditions were optimised based on the trends established for TETA. Membranes were treated with 5% w/v PEI solution for 72 h at room temperature, followed by a 2 h post-heating step at 70 °C. FTIR analysis of PC100 (Fig. 6(b) and (c)) verified the successful attachment of PEI, evidenced by prominent N–H stretching bands at 3200–3400 cm^−1^ and the emergence of urethane-related C=O stretching at 1650–1720 cm^−1^, indicating carbamate bond formation. These spectral changes, coupled with a decrease in contact angle for PC membranes with different pore diameters (Table S5 in SI), signify the introduction of hydrophilic amine moieties on the PC membrane surface. The consistent trends observed across PC100, PC50, and PC15 membranes indicate that the aqueous PEI functionalisation approach is robust, reproducible, and effective even for high-molecular-weight polymeric amines, with the macromolecular PEI chains exhibiting excellent compatibility with sub-100 nm pores and retaining efficient reactivity under nanoscale confinement.

XPS analysis of PEI-functionalised PC100 membranes reveals that functionalisation with 5% w/v resulted in a nitrogen content of 16 at% (see elemental composition in Table S6 in SI), which is substantial yet lower than that obtained with 5% w/v TETA (22.6 at%). PEI, a polymer with a high density of amino groups, was expected to attach to the membranes *via* higher-density amino-group grafting. However, the nitrogen content achieved was lower than that obtained with small molecules, such as TETA. This discrepancy can be attributed to the difficulty of incorporating relatively large polymer chains, such as PEI, into the small nanochannels of PC membranes, compared with smaller molecules such as TETA.^29,30^

To probe the effect of polymer concentration, the PEI loading was increased to 10% w/v, and the reaction was conducted directly at 70 °C for 2 h. With a PEI concentration of 10% w/v, the nitrogen content was enhanced in the PC membranes to 20.3 at% for PC100, 20.2% for PC50, and 19.6% for PC15 (see Fig. S6 and Table S6 in SI), confirming a concentration-dependent enhancement of grafting. Thus, PEI functionalisation demonstrates that polymeric amines can achieve high degrees of surface modification under mild aqueous conditions while maintaining pore morphology. Although an increase in the at% of nitrogen was observed when the PEI concentration was adjusted from 5 to 10% w/v, the nitrogen content (22.6 at%) in the PC membrane with TETA at 5% w/v still exceeded that with PEI at 10% w/v (20.3 at%). This suggests that, for membranes with small pore diameters (15–100 nm), functionalisation with smaller, flexible molecules with high amino-group density, such as TETA, is more effective than functionalisation with bulky polymeric molecules, such as PEI. This outcome reinforces that the molecular size and diffusivity of amines within small nanochannels, rather than the number of reactive amine sites, dominate the effective grafting efficiency in nanodimensional geometries.^31,32^

### Comparison of the positive surface charge density in PC with different functional molecules

**Fig. 7:**
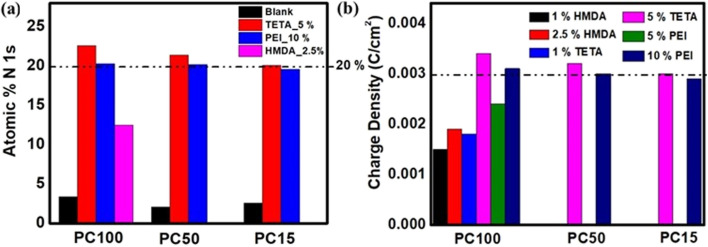
Graph summarising (a) the atomic percentage of nitrogen (N 1s), as determined from XPS analysis, before and after functionalisation of PC100, PC50 and PC15 using HMDA 2.5% w/v, TETA 5% w/v and PEI 10% w/v, (b) the estimated charge densities of functionalised PC100, PC50 and PC15 membranes with amines based on eqn (2)–(4).

To compare the effects of the molecular structures of the functionalised organic molecules on nitrogen incorporation and charge tuning, PC membranes with nominal pore diameters of 100, 50, and 15 nm were functionalised with HMDA, TETA, and PEI at their respective optimal concentrations. Across all pore sizes, amine-functionalised membranes showed substantially higher nitrogen contents (9.7–22.6 at%) than pristine PC (1.5–3.4 at%). Additionally, a shift in the peaks for O 1s and C 1s was also noted in the XPS spectra before and after functionalisation with different amines, suggesting the covalent bonding of amino-containing molecules to PC membranes through urethane bond formation. In batch reactions performed under identical conditions for PC100, PC50 and PC15, TETA (5% w/v) and PEI (10% w/v) yielded *N* = 20.7 ± 1.1 at% for PC15/PC50/PC100, indicating pore-size-independent access within 15–100 nm nanochannels. Fig. 7(a) summarises N 1s at% for the three organic molecules at their highest non-degrading loadings (HMDA 2.5% w/v; TETA 5% w/v; PEI 10% w/v). The observed trend in at% nitrogen obtained, *i.e.* HMDA < PEI ≲ TETA, arises from the interplay between molecular amine functionality, which favours TETA, and steric or diffusional constraints within confined pores, which limit PEI incorporation. Consequently, TETA achieves the highest level of nitrogen functionalisation. Remarkably, a nitrogen content of 20.7 ± 1.1 at% was obtained for PC membranes (across all pore sizes) at their optimal concentrations, among the highest reported for amine-functionalised PC membranes, demonstrating the high efficiency of the applied functionalisation strategy.

In the animation of PC membranes, surface amino groups become protonated under acidic conditions, yielding positively charged ammonium species (–NH_3_^+^, –NH_2_^+^R, –N^+^HR_2_). Unlike quaternary ammonium groups, which are permanently charged and require chemical alkylation, these protonated species are pH-dependent, thereby conferring a tuneable positive surface charge.^33^ Therefore, the presence and proportions of these amino species directly influence the positive surface charge density of the membrane surface. For instance, Mattarozzi *et al.*,^34^ previously reported functionalisation of carbon supports with different alkyl amines to offer enhanced electrolytic reduction of CO_2_ to CO *via* silver nanoparticles. Using XPS, they observed an increase in nitrogen content from 0.5 to 2.1 at% with alkyl amine functionalisation, which correlated with an enhanced positive surface charge in their system.^34^

The correlation between nitrogen grafting density and surface charge is further substantially evidenced by studies in the literature. Zang *et al.*,^35^ developed a novel positively charged membrane, prepared by bio-inspired adhesion of polydopamine (PDA) and surface grafting of poly(ethylene imine) onto polyethersulfone (PES) membrane (PEI-PDA/PES). XPS analysis revealed an increase in nitrogen content from 5.7 at% (PDA/PES) to 13.3 at% (PEI-PDA/PES), confirming successful functionalisation with PEI. This increase in nitrogen content quantified the added amine content in PDA/PES with PEI. It was observed that with the addition of PEI, the zeta potential values shifted from −15.3 ± 0.3 mV (PDA/PES) to +12.1 ± 0.3 mV for PEI-PDA/PES, indicating a remarkably increased positive surface charge density with PEI.

Each nitrogen-containing amine group can contribute a single unit of positive charge in the form of a quaternary ammonium or protonated amino group. Thus, the nitrogen atomic percentage obtained from XPS serves as a reliable indicator of grafting density and the corresponding surface charge density. Table 2 provides a detailed estimation of the grafting densities and the corresponding surface charge densities for the highest nitrogen percentages (nitrogen at%) achieved with different amines, based on XPS analysis, estimated from eqn (2)–(4). By combining the atomic percentage of nitrogen with the total nuclear density, the number of nitrogen atoms per square centimetre (nitrogen atoms cm^−2^) was calculated (eqn (2)). This value was then used to determine the grafting density (*ρ*, eqn (3)). Notably, the smaller amine molecules, HMDA and TETA, demonstrated higher *ρ* than the larger polymeric amine, PEI, with HMDA achieving the highest *ρ*. This trend indicates that grafting efficiency is governed not only by nitrogen content but also by the molecular size of the functionalising agents. The enhanced accessibility and diffusivity of smaller amines within the confined nanochannels of the membranes account for this trend.^29,30^

**Table 2:** Estimated grafting density and charge density calculated from N at% obtained from XPS analysis^a^

Sample	Est. N per molecule	N at% (XPS)	N (at. cm^−2^)	Grafting density (mol cm^−2^)	Charge density (C cm^−2^)
PC100-HMDA	2	12.5	1.2 × 1016	9.9 × 10^−9^	0.0019
2.5% w/v					
73 h RT					
PC100-TETA	4	22.6	2.1 × 1016	8.8 × 10^−9^	0.0034
5% w/v					
2 h, 70 °C					
PC100-PEI	14	20.3	1.9 × 1016	2.3 × 10^−9^	0.0030
0.8 KD					
10% w/v					
2 h, 70 °C					

a PEI-0.8 KD, *M*_*n*_ ∼600 g mol^−1^, Est. *N* ∼14.

Fig. 7(b) compares the calculated charge densities in PC membranes of different nominal pore sizes functionalised with different amine-containing molecules. For all the studied amines, the maximum nitrogen atomic percentage was observed with TETA on PC-100 membranes (22.6 at%). Assuming that all grafted nitrogen atoms are fully protonated to form quaternary ammonium groups, the calculated nitrogen atom density (nitrogen atoms cm^−2^) can be directly correlated with the resulting surface charge density (*σ*). The highest *σ* of 0.0034 C cm^−2^ was achieved using 5% w/v TETA on PC-100 membranes (Table 2 and Fig. 7(b)). In contrast, HMDA at 1% w/v, which yielded the lowest nitrogen atomic percentage (9.7 at%) among all functionalised membranes, corresponded to a *σ* of 0.0015 C cm^−2^. These nitrogen percentages and the corresponding estimated charge densities are among the highest reported for PC membranes functionalised under aqueous conditions, while remaining compatible with ultrasmall (∼15 nm) pores.^19,36,37^ This underscores the efficiency of the aqueous, low-temperature functionalisation strategy, employing a broad range of amines, to achieve tuneable surface charge densities spanning 0.0015–0.0034 C cm^−2^. A broader comparison with representative literature systems is provided in Table 3, highlighting that the present aqueous functionalisation yields^1–10,12–39^ nitrogen at% and *σ* values than previous functionalisation methods in PC and polymer-based systems.

**Table 3:** Comparison of nitrogen content (N at%), surface zeta potential, and estimated surface charge density (*σ*) for amine-functionalised and cationic polymer-modified membranes, including this work and representative literature reports. Charge density (*σ*) is estimated for literature references with the N at% based on eqn (2)–(4)

Membrane (substrate)	Modification condition	N at%	Zeta potential (mV)	Charge density (C cm^−2^)
PC-HMDA [This work]	Aqueous-based, 2.5% w/v, RT	12.5	+15 to +20 (expected)	0.0019
PC-TETA [This work]	Aqueous-based, 5% w/v, 70 °C	22.6	+20 to +30 (expected)	0.0034
PC-PEI (0.8 KD) [This work]	Aqueous-based, 10% w/v, 70 °C	20.3	+15 to +25 (expected)	0.0030
PC-HMDA^19^	Aqueous-based, 1% w/v, RT	6.4	Not reported	< 0.001 (est.)
Polyethersulfone (PES)^35^	Bio-inspired adhesion of polydopamine + PEI grafting in aqueous solution @ 27–80 °C	13.3	+4.6 to +16.5 for PEI-grafted PDA/PES membranes, enhanced from −41 for pristine PES substrate	∼0.002 (est.)
PVDF(Polyvinylidene fluoride)-poly(amidoamine) (PAMAM)^38^	Simple alkaline pretreatment followed by anchoring PAMAM	4.2–7.6 (depending on dendrimer generation)	After PAMAM grafting, zeta potential is slightly increased to positive side (−61.5 mV to −59.2 mV @ pH 8.7	∼0.001–0.002 (est)
Plasma-induced poly(2-aminoethyl methacrylate) (AEMA) post-modification of PC^14^	Plasma induced modification	6.2 for PC-AEMA	Not reported	∼0.001–0.002 (est.)

### Functionalisation of PC membrane with glycine for negative charge grafting

**Fig. 8:**
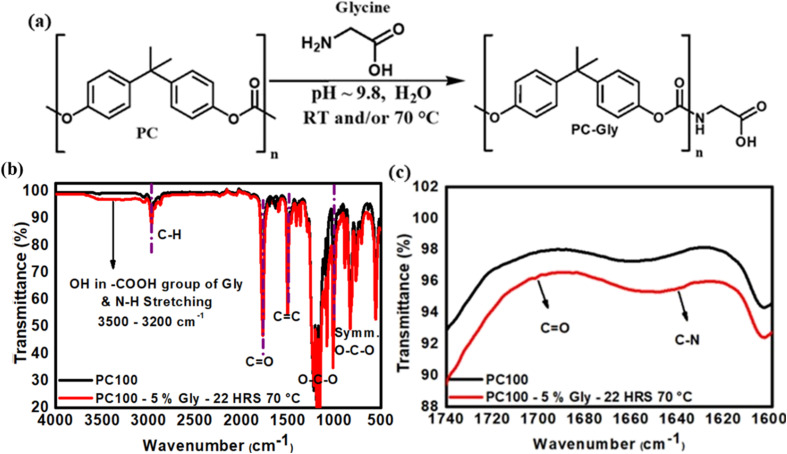
(a) Scheme representation of the chemical derivatisation of PC membrane with glycine (Gly). (b) FTIR spectra of PC100 membranes before and after functionalisation with Gly 5% w/v (indicating the characteristic pristine PC peaks), highlighting (c) the carbonyl group (C=O) region 1600–1750 cm^−1^, showing slight shifts indicative of urethane bond formation between Gly and PC.

Building on the positive charge modulation achieved by amine grafting, we further investigated the generation of negative surface charge using glycine (Gly), the simplest zwitterionic amino acid, which contains both an amine and a carboxylic acid group. During grafting, the amine moiety of glycine reacts with the carbonate backbone of the PC membrane, while the carboxylic acid group remains available at the surface. Under neutral to basic conditions, this carboxylic group readily deprotonates to form a negatively charged carboxylate (–COO^−^), thereby imparting a stable negative surface charge to the membrane.^39^ A reaction scheme analogous to the earlier amination route for grafting a positive charge was employed for PC membrane functionalisation with glycine. In this functionalisation method, the amine end of glycine attacks the electrophilic carbonate carbon of the PC backbone, forming a urethane linkage, anchoring the molecule, and exposing the free –COOH/–COO^−^ group at the interface (Fig. 8(a)).

For glycine (Gly), the initial functionalisation using a 5% w/v solution for 72 h at RT, followed by an additional 2 h at 70 °C did not produce notable changes in either contact angle measurements or in FTIR spectra, suggesting sub-optimal grafting conditions. Consequently, the reaction parameters were modified, and the process was carried out at 70 °C for 22 h to enhance surface interactions and grafting. Given that glycine contains carboxylic acid and a primary amine (–NH_2_) group, the solution pH was adjusted above glycine's second p*K*_a_ value (>9.6) to ensure the amine remained unprotonated and the carboxyl group deprotonated, promoting a net negative surface charge. FTIR spectra of glycine-functionalised PC100 (Fig. 8(b) and (c)) displayed new peaks at 3200 to 3500 cm^−1^ (O–H and N–H stretching) and 1705 cm^−1^ (C=O stretch in carboxyl), confirming successful attachment of glycine on PC. Similar observations in the FTIR (new peaks between 3200 and 3500 cm^−1^, indicating O–H and N–H stretching) also confirm the attachment of Gly to PC50 and PC15 (see Fig. S7 in the SI).

**Fig. 9:**
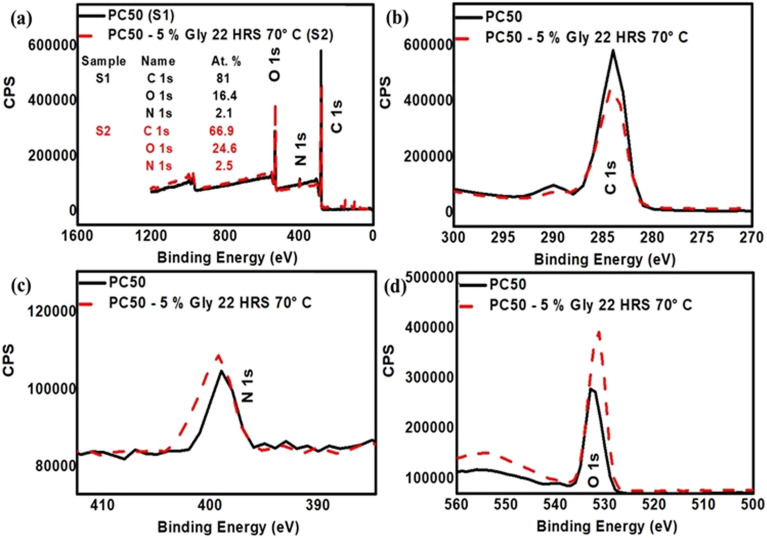
(a) XPS survey spectra of PC50 membranes before and after functionalisation with Gly (5% w/v, 22 h, 70 °C), showing the atomic percentages of C 1s, O 1s, and N 1s. High-resolution spectra of the (b) C 1s (270–300 eV), (c) N 1s (380–420 eV), and (d) O 1s (520–545 eV) regions reveal an increased N 1s signal and shifts in the C 1s and O 1s peaks, confirming successful Gly grafting onto the PC membranes.

XPS analysis was conducted to quantify the nitrogen content in glycine-functionalised PC membranes. The corresponding elemental compositions for PC100, PC50, and PC15 membranes are summarised in Table S7 (see SI), confirming successful incorporation of nitrogen species following glycine grafting. For PC100, the nitrogen content is 2.3 at% following glycine functionalisation (see SI, Table S7). In contrast, the oxygen content increased from around 14.8 at% in the untreated membranes to 16.3 at% in the PC100-glycine membranes, indicating the incorporation of glycine's carboxylic acid group. Fig. 9 presents the XPS spectra of representative PC50 membranes before and after functionalisation with 5% w/v glycine for 22 h at 70 °C, indicating increased N and O contents. Similar increases in both nitrogen and oxygen contents were observed across membranes PC100, PC50 and PC15, confirming consistent grafting efficiency and chemical modification throughout the PC membrane series (Fig. 9(c), (d), and Table S7, in SI). Having established both cationic (amine-based) and anionic (carboxylate-based) surface modifications on track-etched polycarbonate, this work demonstrates that surface charge polarity and density can be precisely tuned by molecular design and reaction conditions.

## Conclusion

This work establishes an aqueous strategy for tuning the surface charge of track-etched polycarbonate (PC) membranes through controlled molecular functionalisation. Membranes with nominal pore sizes of 15–100 nm were successfully grafted with amine- and amino acid-containing molecules, thereby enabling modulation of both positive and negative charges. FTIR and XPS confirmed covalent attachment *via* a urethane linkage, while systematic variations in temperature, concentration, and molecular structure enabled precise control of nitrogen incorporation and the resulting charge density.

Among the studied modifiers, triethylenetetramine (TETA) achieved the highest nitrogen incorporation (22.6 at%) and corresponding surface charge density (0.0034 C cm^−2^), outperforming both hexamethylenediamine (HMDA) and polyethyleneimine (PEI). The enhanced grafting efficiency of TETA arises from its greater amine functionality and molecular mobility relative to sterically hindered PEI. Conversely, glycine functionalisation introduced carboxylate-terminated surfaces with increased oxygen content, thereby enabling the successful generation of negatively charged PC membranes under alkaline conditions. Collectively, these results reveal clear structure–reactivity relationships: smaller, multifunctional amines maximise nitrogen uptake and positive charge density, whereas compact amino acids enable the introduction of stable negative charge. The ability to achieve bidirectional, both positive and negative, surface functionalisation under mild, aqueous conditions establishes a foundation for developing polycarbonate as a versatile nanofluidic membrane platform capable of supporting both ion-selective and charge-governed transport processes.

The developed aqueous modification route avoids harsh solvents, high-energy plasma, or UV processes while achieving nitrogen levels and charge densities that rival or exceed those of previously reported methods. This scalable, low-energy functionalisation protocol thus provides a sustainable pathway for fabricating charge-tuneable PC membranes suitable for advanced applications in ion-selective separation, nanofluidic energy conversion, thermoelectric systems, and electrochemical sensing. More broadly, the methodology can be extended to other polymeric or bio-derived membranes to achieve tailored interfacial charge profiles, establishing a general framework for surface-charge-governed transport in next-generation energy and environmental technologies.

## Author contributions

Anjali Ashokan: conceptualisation, methodology, investigation, formal analysis, validation, writing – original draft, and writing – review & editing. Kamil Rahme: conceptualisation, methodology development, investigation, formal analysis, validation, writing – original draft. Subhajit Biswas: formal analysis, writing – review & editing, supervision, validation. Justin D. Holmes: formal analysis, writing – review & editing, supervision, resources, validation. All authors have read and approved the final version of the manuscript.

## Conflicts of interest

There are no conflicts to declare.

## Supplementary Material

RA-015-D5RA06556A-s001

## Data Availability

The datasets generated during and/or analysed during the current study are available in the TRANSLATE Zenodo repository, https://zenodo.org/communities/translateh2020/. Supplementary information (SI): additional materials characterisation data for pristine and aminated track-etched polycarbonate membranes, including photographic images and SEM pore-size analysis, FTIR spectra before and after functionalisation, and water contact angle measurements. It also contains XPS survey and high-resolution spectra with corresponding elemental compositions, confirming successful HMDA, TETA, PEI and glycine grafting and quantifying nitrogen incorporation across different pore sizes and treatment conditions. See DOI: https://doi.org/10.1039/d5ra06556a.
