# Supplementary material for: Aqueous amination of track-etched polycarbonate membranes for tuneable nanochannel surface charge density
Source: RSC Adv. 2025 Dec 5;15(56):48247–60. doi: 10.1039/d5ra06556a (PMC12681244; doi:10.1039/d5ra06556a)
Supplement: RA-015-D5RA06556A-s001 [file RA-015-D5RA06556A-s001.pdf]

Supplementary Information (SI) for RSC Advances.
This journal is © The Royal Society of Chemistry 2025

Supporting Information

Aqueous Amination of Track-Etched Polycarbonate Membranes for Tuneable Nanochannel Surface Charge Density

Anjali Ashokan, Kamil Rahme, Subhajit Biswas* and Justin D. Holmes*

School of Chemistry, University College Cork, Cork, T12 YN60, Ireland.

AMBER Centre, Sustainability Institute, University College Cork, Cork, T23 XE10, Ireland.

*Corresponding authors: j.holmes@ucc.ie & s.biswas@ucc.ie

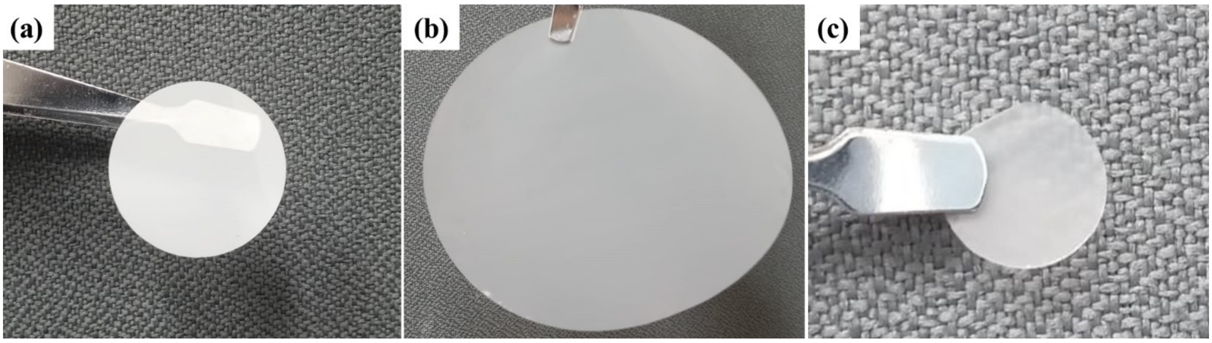

Figure S1. Photographic images of Isopore™ Merck Millipore membranes with nominal pore sizes of (a) 0.1 μm, (b) 0.05 μm, and (c) 0.015 μm.

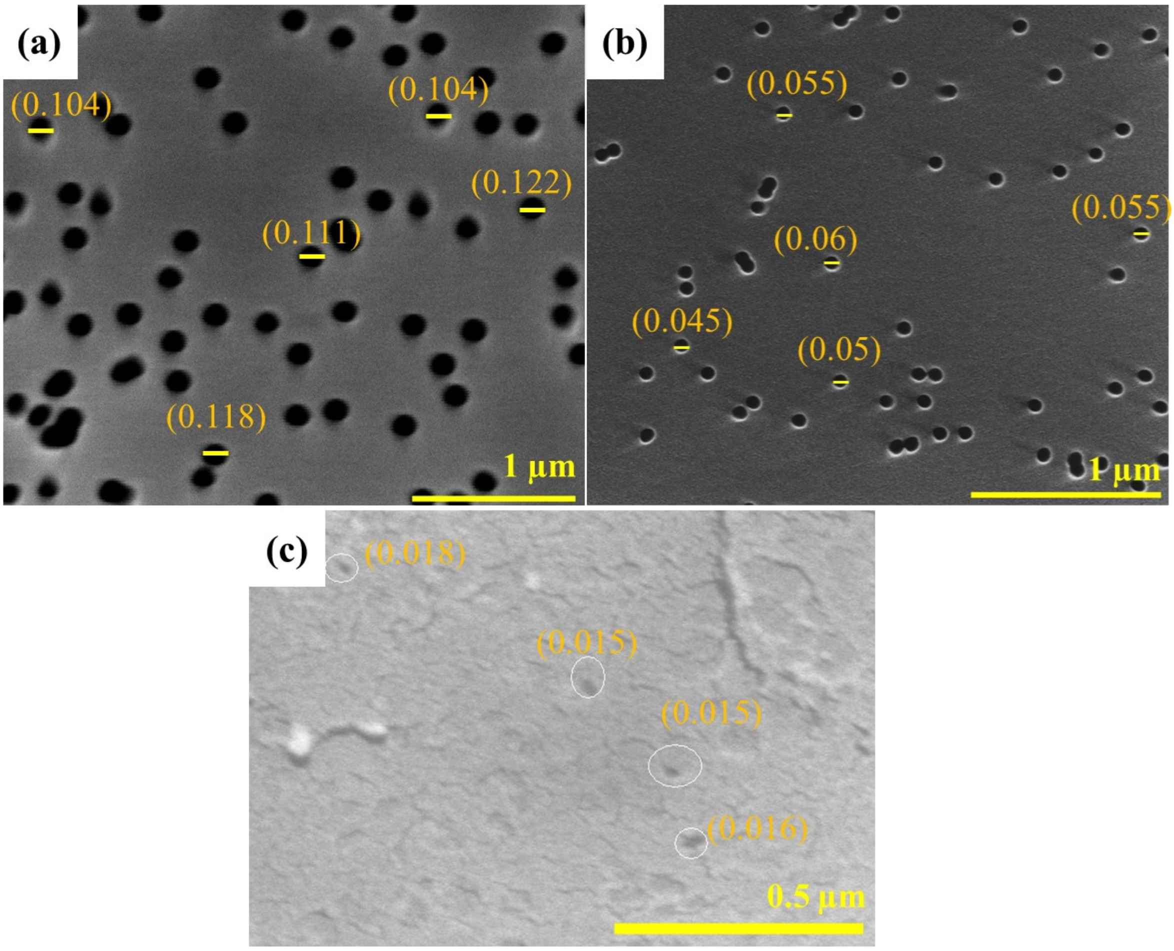

Figure S2. SEM images of Isopore™ Merck Millipore membranes with pore sizes of (a) 0.1 µm, (b) 0.05 µm, and (c) 0.015 µm, analysed using ImageJ software.

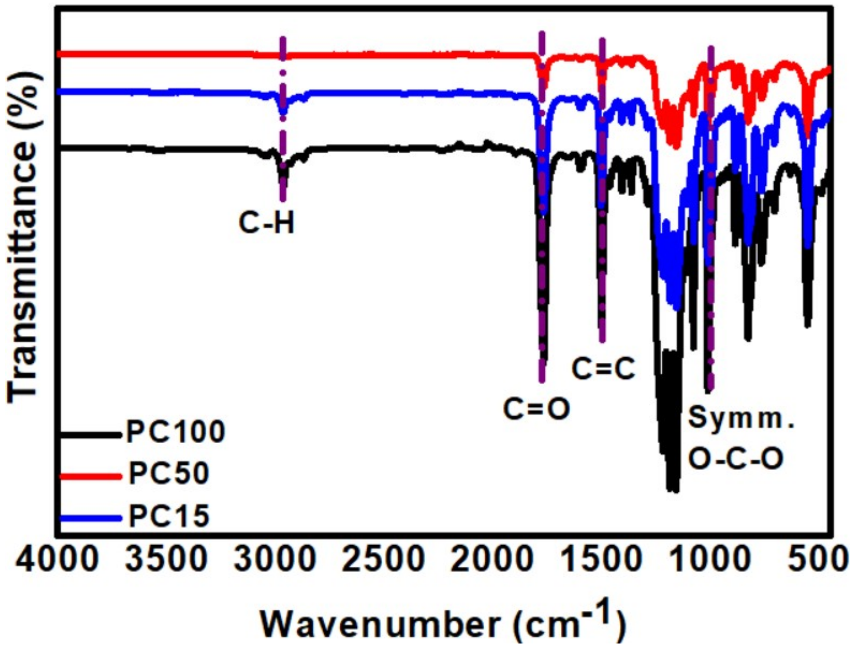

Figure S3. FTIR spectra of pristine PC100, PC50, and PC15 membranes.

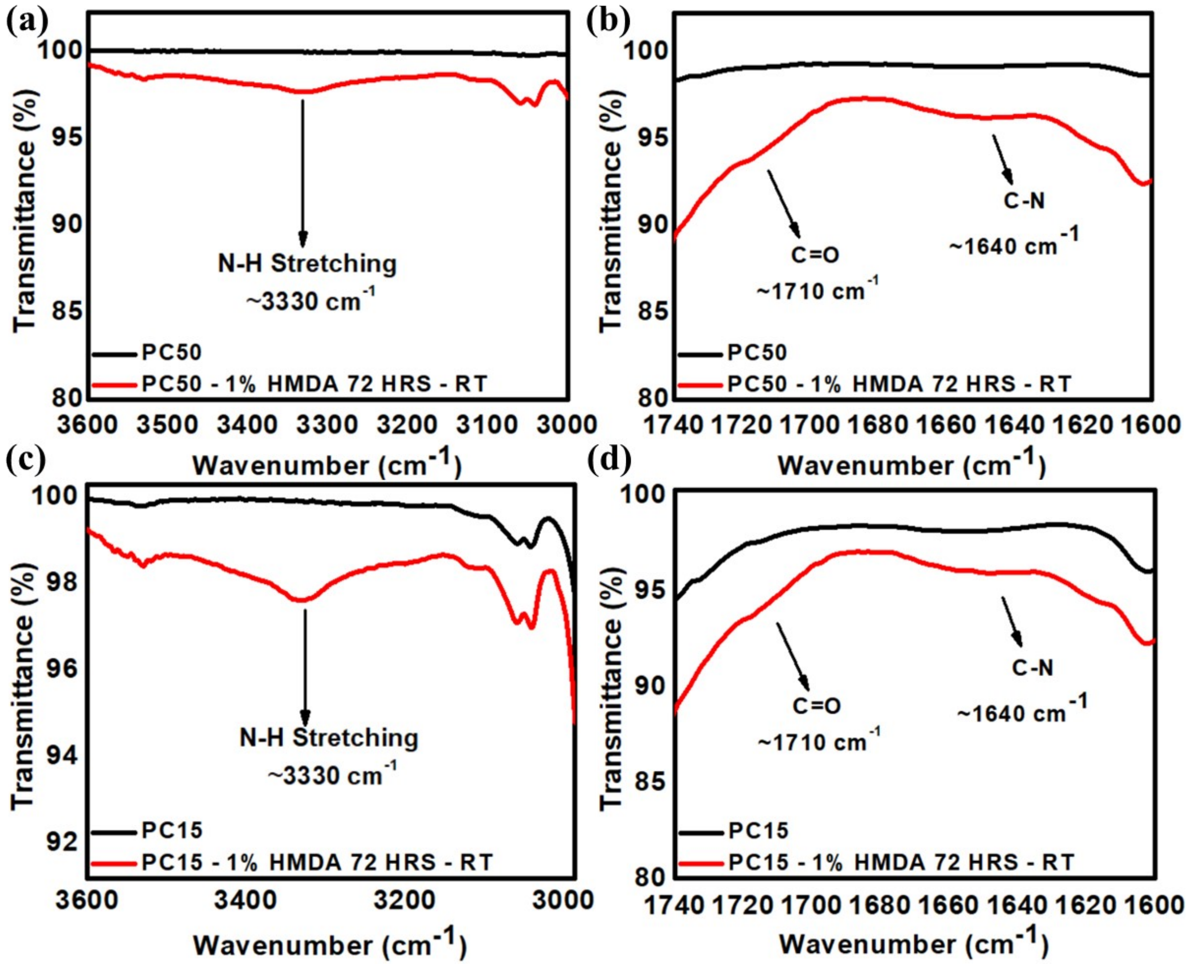

Figure S4. FTIR spectra of (a), (b) PC50 and (c), (d) PC15 membranes before and after functionalisation with 1 % w/v HMDA, highlighting (i) the 3000-3600 cm^{-1} region, where N-H stretching bands from HMDA appear, and (ii) the 1600-1740 cm^{-1} carbonyl (C=O) region, where slight shifts indicate urethane bond formation between HMDA and PC.

Table S1. Contact angles of PC membranes (0.1, 0.05, and 0.015 μm) were measured for untreated (blank) samples and for those treated with 1 % w/v HMDA for 72 h at room temperature.

Membrane	Mean Contact Angle (°)
100 nm pore size:<br>- PC100-BLANK<br>- PC-100-HMDA, 1 % w/v, 72 h at RT	 71.7<br>91.7
50 nm pore size:<br>- PC50-BLANK<br>- PC-50-HMDA, 1 % w/v, 72 h at RT	 67.8<br>95.3
15 nm pore size:<br>- PC15-BLANK<br>- PC-15-HMDA, 1 % w/v, 72 h at RT	 64.5<br>81.2

Table S2. Contact angles of PC membranes (0.1, 0.05, and 0.015 µm) for untreated (blank) samples and for those treated with 1 % w/v TETA for 74 h at room temperature, followed by heating at 70 °C for 2 h.

**Membrane**	**Mean Contact Angle (°)**
100 nm pore size:<br>- PC100-BLANK<br>- PC-100-TETA, 1 % w/v, 74 h at RT<br>- PC-100-TETA, 1 % w/v, 74 h at RT, then 2 h at 70 °C	 64.6<br>66.11<br>56.4
50 nm pore size:<br>- PC50-BLANK<br>- PC-50-TETA, 1 % w/v, 74 h at RT<br>- PC-50-TETA, 1% w/v, 74 h at RT, then 2 h at 70 °C	 67.8<br>67.8<br>55.9
15 nm pore size:<br>- PC15-BLANK<br>- PC-15-TETA, 1 % w/v, 74 h at RT<br>- PC-15-TETA, 1 % w/v, at 74 h at RT, then 2 h at 70 °C	 64.5<br>63.5<br>51.4

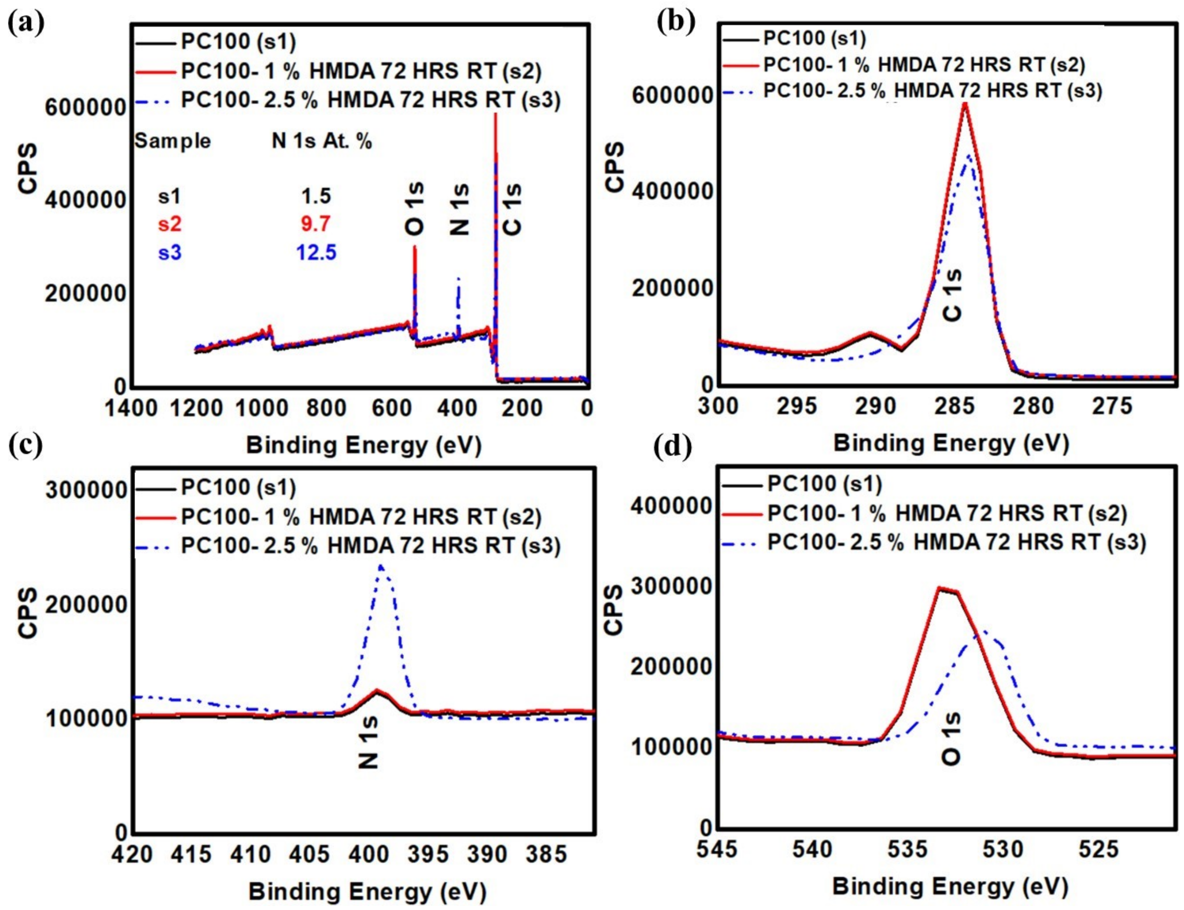

Figure S5. (a) XPS survey spectra of PC100 membranes before and after functionalisation with 1 % w/v HMDA (72 h at RT) and 2.5 % w/v HMDA (72 h at RT), with N 1s atomic % indicated. High-resolution spectra of (b) C 1s (270-300 eV), (c) N 1s (380-420 eV), and (d) O 1s (520-545 eV) regions show increased N 1s signal and shifts in C 1s and O 1s peaks, confirming successful HMDA grafting onto PC membranes.

Table S3. Summary of elemental quantification obtained from XPS survey spectra, for HMDA functionalised PCs.

Sample	Atomic %						
	**O 1s**	**C 1s**	**N 1s**	**S 2p**	**Cl 2p**	**Na 1s**	**Si 2p**
PC100-Blank	14.8	81.6	3.4	0.2	-	-	-
PC100-HMDA,<br>1 % w/v, 72 h at RT	12.5	77.7	9.7	0.1	0.1	-	-
PC100-HMDA,<br>2.5 % w/v, 72 h at RT	11.3	76.1	12.5	0.1	-	-	-

Table S4. Summary of elemental quantification obtained from XPS survey spectra, for TETA functionalised PCs.

Sample	Atomic %						
	**O 1s**	**C 1s**	**N 1s**	**S 2p**	**Cl 2p**	**Na 1s**	**Si 2p**
PC100-Blank	14.8	81.6	3.4	0.2	-	-	-
PC100-TETA, 1 % w/v, 74 h at RT + 2 h at 70 °C	14.5	72.3	12.1	0.1	0.4	0.6	-
PC100-TETA, 5 % w/v, 2 h at 70 °C	13.1	64.0	22.6	0.1	0.2	-	-
PC-50-Blank	16.4	81.0	2.1	0.3	0.3	-	-
PC50-TETA, 5 % w/v, 2 h at 70 °C	13.9	64.0	21.4	0.1	0.4	0.1	-
PC15 Blank	16.0	80.5	2.6	0.2	0.7	-	-
PC15-TETA, 5 % w/v, 2 h at 70 °C	14.5	64.8	20.1	0.1	0.3	0.2	-

Table S5. Contact angles of PC membranes (0.1, 0.05, and 0.015 μm) for untreated (blank) samples and for those treated with 5 % w/v PEI (0.8 kDa) for 72 h at room temperature, followed by heating at 70 °C for 2 h.

Membrane	Contact Angle Value (°)
100 nm pore size:	
- PC100-BLANK	68.4
- PC-100-PEI, 72 h at RT	63.3
- PC-100-PEI, 72 h at RT + 2 h at 70 °C	48.6
50 nm pore size:	
- PC50-BLANK	67.8
- PC-50-PEI, 72 h at RT	58.2
- PC-50-PEI, 72 h at RT + 2 h at 70 °C	36.1
15 nm pore size:	
- PC15-BLANK	64.5
- PC-15- PEI, 72 h at RT	59.5
- PC-15-PEI, 72 h at RT + 2 h at 70 °C	34.3

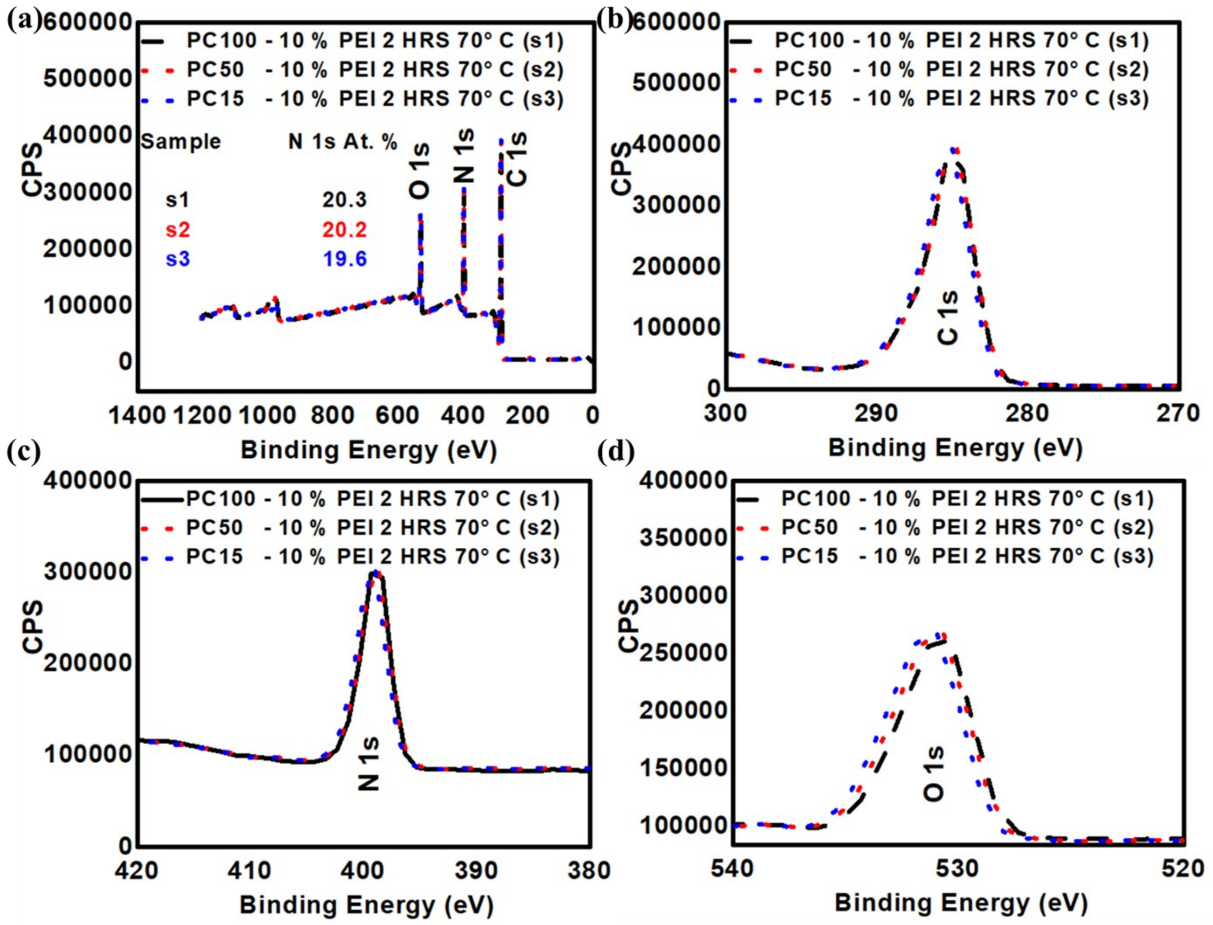

Figure S6. (a)XPS survey spectra of PC100, PC50 and PC15 membranes before and after functionalisation with 10 % w/v PEI 800D (72 h at 70 °C), with N 1s atomic % indicated. High-resolution spectra of (b) C 1s (270-300 eV), (c) N 1s (380-420 eV), and (d) O 1s (520-545 eV) regions.

Table S6. Summary of elemental quantification obtained from XPS survey spectra, for PEI functionalised PCs.

Sample	Atomic %						
	**O 1s**	**C 1s**	**N 1s**	**S 2p**	**Cl 2p**	**Na 1s**	**Si 2p**
PC100-PEI, 5 % w/v, 72 h at RT + 2 h at 70 °C	13.3	70.5	16.0	0.1	0.1	-	-
PC100-PEI, 10 % w/v, 2 h at 70 °C	12.9	66.3	20.3	0.1	0.2	0.1	-
PC50-PEI, 10 % w/v, 2 h at 70 °C	13.8	65.0	20.2	0.1	0.5	0.3	-
PC15-PEI, 10 % w/v, 2 h at 70 °C	13.5	65.9	19.6	0.1	0.6	0.2	-

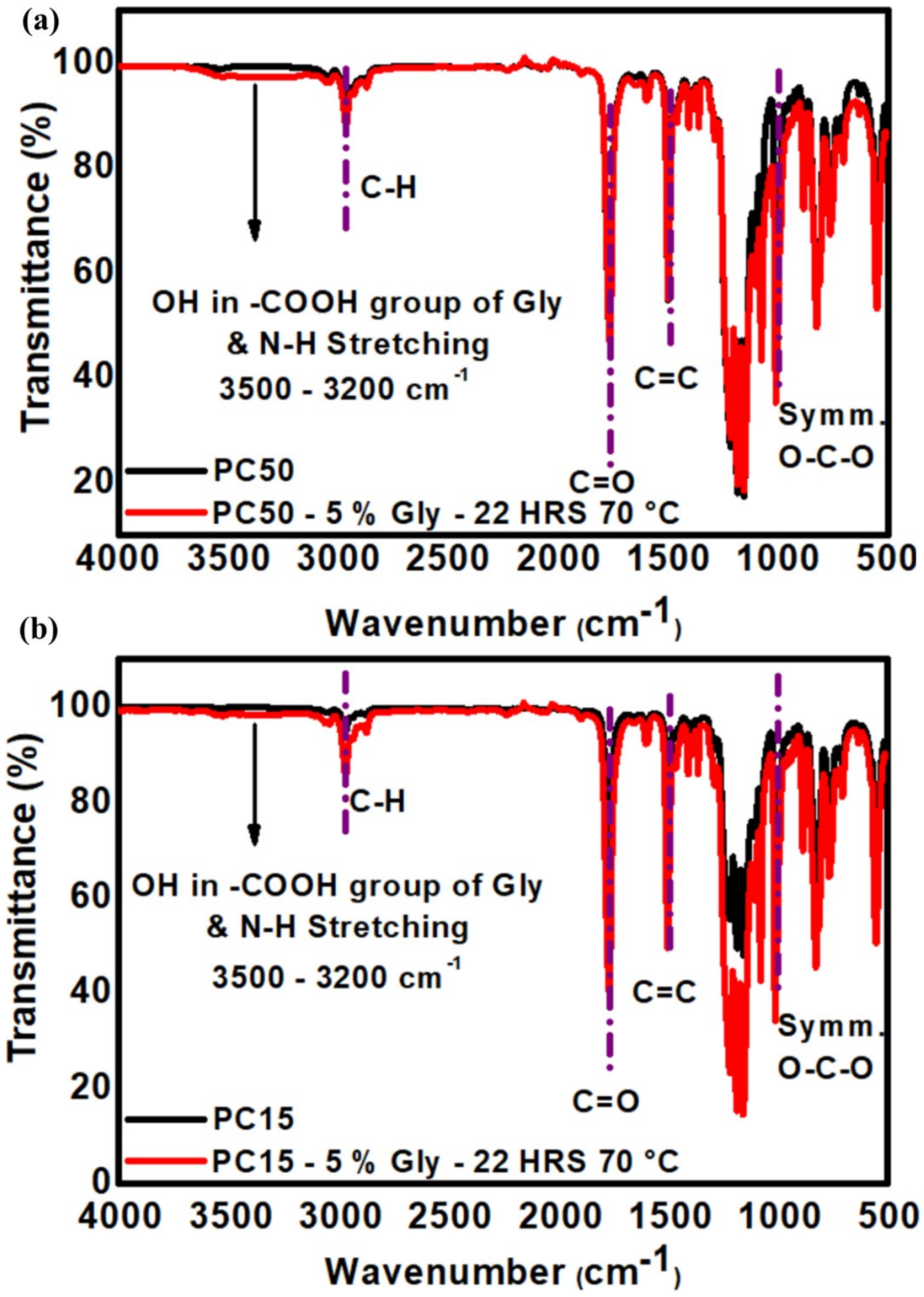

Figure S7. FTIR spectra of (a) PC50 and (b) PC15 membranes before and after functionalisation with Gly 5 % w/v.

Table S7. Summary of elemental quantification obtained from XPS survey spectra, for GLY functionalised PCs.

**Sample**	**Atomic %**						
	**O 1s**	**C 1s**	**N 1s**	**S 2p**	**Cl 2p**	**Na 1s**	**Si 2p**
PC100-Glycine, 1 % w/v, 72 h at RT + 2 h at 70 °C	16.1	81.4	2.2	0.1	-	-	0.2
PC100-Glycine, 5 % w/v, 22 h at 70 °C	16.3	81.0	2.3	0.1	0.3	-	-
PC50-Glycine, 5 % w/v, 22 h at 70 °C	24.6	66.9	2.5	-	4.8	-	-
PC15-Glycine, 5% w/v, 22 h at 70 °C	16.7	80.0	2.2	0.2	0.7	0.1	0.2
